# Reward Guides Vision when It's Your Thing: Trait Reward-Seeking in Reward-Mediated Visual Priming

**DOI:** 10.1371/journal.pone.0014087

**Published:** 2010-11-23

**Authors:** Clayton Hickey, Leonardo Chelazzi, Jan Theeuwes

**Affiliations:** 1 Department of Cognitive Psychology, VU University Amsterdam, Amsterdam, The Netherlands; 2 Department of Neurological and Visual Sciences, University of Verona, Verona, Italy; 3 National Institute of Neuroscience, Verona, Italy; University of Sydney, Australia

## Abstract

Reward-related mesolimbic dopamine is thought to play an important role in guiding animal behaviour, biasing approach towards potentially beneficial environmental stimuli and away from objects unlikely to garner positive outcome. This is considered to result in part from an impact on perceptual and attentional processes: dopamine initiates a series of cognitive events that result in the priming of reward-associated perceptual features. We have provided behavioural and electrophysiological evidence that this mechanism guides human vision in search, an effect we refer to as reward priming. We have also demonstrated that there is substantial individual variability in this effect. Here we show that behavioural differences in reward priming are predicted remarkably well by a personality index that captures the degree to which a person's behaviour is driven by reward outcome. Participants with reward-seeking personalities are found to be those who allocate visual resources to objects characterized by reward-associated visual features. These results add to a rapidly developing literature demonstrating the crucial role reward plays in attentional control. They additionally illustrate the striking impact personality traits can have on low-level cognitive processes like perception and selective attention.

## Introduction

Reward signals encoded in mesolimbic dopamine are thought to guide animal approach behavior, biasing animals towards objects associated with prior reward and away from objects associated with sub-optimal outcome. Some theories of dopamine, like the *incentive salience hypothesis* of Berridge and Robinson [1], suggest that this is instantiated in biases of perception and attention. The idea is that release of mesolimbic dopamine causes a chain of events that leads to facilitated processing of reward-conditioned perceptual features. Attention is thus guided to environmental stimuli that are likely to garner positive outcomes [1]–[5].

This framework has been influential in the reinforcement learning literature and has been applied to human behavior in clinical settings like addiction research [6], but has not been widely adopted in the psychological investigation of attention (though see [7], [8]). Psychological and neuroscientific models of attentional control often characterize attention as under the combined influence of exogenous factors, directing attention towards salient stimuli, and endogenous factors, biasing attention towards task-relevant stimuli [9], [10]. Reward's role in this framework is generally seen as an indirect influence on the strategic establishment of top-down set.

**Figure 1:**
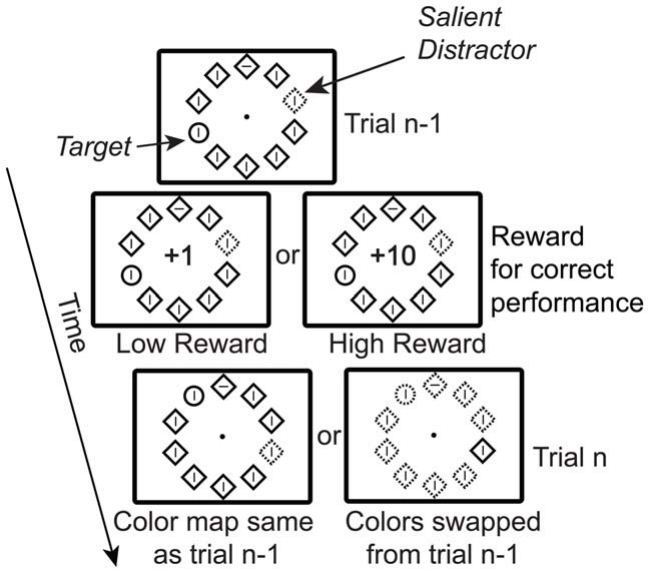
General paradigm. Target and salient distractor denoted.

We have recently reported results from a series of experiments that suggest this perspective greatly underestimates the impact reward can have on visual attention [11], [12]. We had participants complete visual search experiments based on the *additional singleton paradigm* of Theeuwes [13] (see Figure 1). In this type of task participants search for a uniquely shaped target - known in the literature as a *shape singleton -* presented among a number of homogenous distractors. The target is sometimes the only unique object in the search array, but in other trials a *color singleton* is defined by giving one of the distractors unique color (often red when all other stimuli are green or vice versa). The pervasive finding is that participants are slower to discriminate features of the shape singleton target when the color singleton distractor is present in the display, and this has been linked to the capture of attention to the location of the color singleton [13]–[15] (note that there is ongoing debate regarding this issue, see [16], [17] for recent reviews). We modified this paradigm slightly, adding high-magnitude (10 points) or low-magnitude (1 point) reward feedback at the end of every correct trial. Participants were instructed to maximize the number of points they received and were paid based on this number, but in fact reward magnitude was not tied to performance in any way: so long as participants responded correctly, they were as equally likely to receive high-magnitude reward as low.

**Figure 2:**
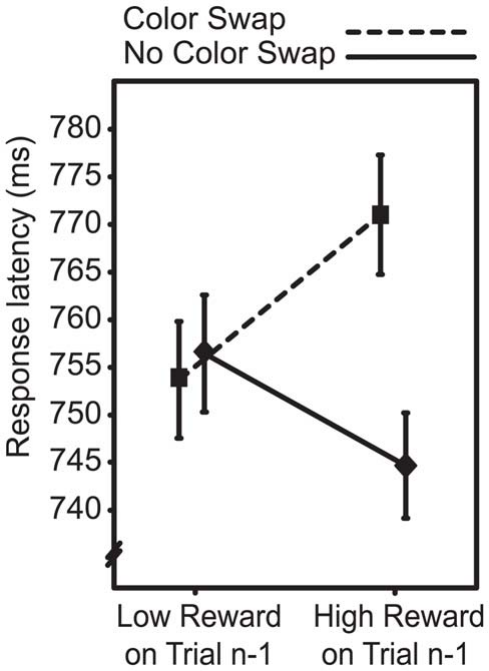
Behavioural results from the visual search task. Error bars reflect within-subject 95% confidence intervals (Cousineau, 2005).

Our analysis centered on two features of the experimental design. First, the colors that defined the target in any given trial could either be the same as those in the previous trial (as when the target was red and the unique distractor was green in both trial n and trial *n*-1), or could have swapped (as when the target was red and the unique distractor was green in trial *n* but the target was green and the unique distractor was red in trial *n*-1; see also [18]). Second, participants could receive either high or low-magnitude reward following each trial. Our expectation was that high-magnitude reward would facilitate subsequent processing of the features that defined the target such that attention was biased towards these features in the next trial. Participants should therefore respond quickly when the same color characterizes the target as did so in the preceding trial. In contrast, when the colors swap, the color associated with reward will come to define the distractor. As a result, the likelihood of attention being captured to the distractor location should increase and reaction times (RTs) should become slower. This pattern was borne out in the data and - to foreshadow - is replicated in the present study (see Figure 2). Importantly, this does not appear to reflect a strategic propensity; we find that subjects erroneously select objects characterized by reward-conditioned features even when a much better strategy is available to them [11, Exp 1]. We refer to this automatic bias as *reward priming*.

We have conducted an event-related potential (ERP) study of reward priming [11, Exp 2], and results from this work confirmed our notion that perceptual and attentional processing of objects characterized by reward-associated visual features is facilitated. We found that the magnitude of an early-latency index of visual perception – the lateralized P1 [19] – was larger in in response to a singleton defined by a color associated with high-magnitude reward, and that a later index of attentional selection - the N2pc [19] – was elicited by the object characterized by the reward-associated color. These effects were observed in response to the stimulus characterized by the reward-associated color regardless of task relevance. This last point bears repetition: perceptual and attentional processing of the target was facilitated when the target was characterized by the color linked to high-magnitude reward in the preceding trial, whereas perceptual and attentional processing of the unique distractor was facilitated when this object was defined by the reward-associated color. No corresponding effects were observed in response to low-magnitude reward feedback.

Our electrophysiological work garnered an additional outcome: the magnitude of an anterior ERP component elicited by high-magnitude reward feedback – the medial frontal negativity (MFN; [20]) - predicted the size of the reward priming effect on a per-subject basis. Consistent with prior source analysis [20], we localized the MFN to anterior cingulate cortex (ACC) and showed that participants with increased reward-related activity in this brain area were more strongly biased towards reward-conditioned visual features. This led us to suggest that participants who show strong reward priming do so because they are especially sensitive to the motivational impact of reward. To clarify, we thought that people who showed greater reward-related activity in ACC might be those with reward-seeking personalities, and that these people may show an increased propensity to attend to stimuli characterized by reward-associated features.

The current study was designed to test this hypothesis. We used a personality inventory to measure trait reward-seeking in participants before having them complete the search task described above. Our expectation was that those participants who show greater trait propensity to reward-driven behavior would be those who show a larger reward priming effect.

The personality inventory we used was the *Behavioral Inhibition System/Behavioral Activation System scale* (BIS/BAS) of Carver and White [21]. This scale was developed based on the theoretical work of Gray [22], [23], who proposed that affect and behavior could be best understood as the outcome of two neurological systems (or perhaps three: Gray suggested the presence of a third *fight or flight system* [23], but often placed theoretical emphasis on the BIS and BAS). The BIS is thought to be largely instantiated in the septohippocampal system, with input from prefrontal cortex, noradrenergic output through the locus coeruleus, and serotonergic output through the median raphe [22], [24]–[26]. High BIS scores have been associated with traits like anxiety and neuroticism, and the system is thought to be generally responsible for inhibition and the establishment of control in response to punishment, sub-optimal outcome, fear, and novelty [21]. In contrast, the BAS is thought to be instantiated in dopaminergic structures, including mesencephalic nuclei like the substantia nigra and ventral tegmental area, and dopaminergic target sites in the basal ganglia, thalamus, and cortex [22], [24]–[26]. High BAS scores suggest extraversion, impulsivity, novelty seeking, and positive affect [21]. Importantly, Gray proposed that the fundamental responsibility of the BAS system is the initiation of reward-seeking behavior [25], [27].

Carver and White's [21] inventory of BIS/BAS sensitivity is a 24-item questionnaire in which participants indicate the degree to which they agree with simple statements (e.g., “I go out of my way to get the things I want”). Large-sample factor analysis has identified two primary dimensions in the results, corresponding to BIS and BAS sensitivity, and three BAS subdimensions: BAS_drive_, BAS_fun seeking_, and BAS_reward responsiveness_ [21]. Two of the three BAS subscales - BAS_drive_ and BAS_reward responsiveness_ – index reward processing personality traits (with BAS_fun seeking_ reflecting something akin to trait novelty-seeking). However, an important distinction needs to be made between these subscales. BAS_reward responsiveness_ indexes the degree to which a person derives pleasure from reward; a person scoring high on this measure might particularly enjoy a fine wine. In contrast, BAS_drive_ captures the strength with which reward outcome guides subsequent behavior; prior experience of a good wine might drive a person scoring high on this measure to develop a fine wine cellar (or rob a liquor store) [21], [30]. There is a clear relationship between these constructs, and the corresponding measures correlate accordingly, but factor analysis demonstrates that they are discrete: some individuals are strongly motivated to behave in a manner that garners good outcome without showing a corresponding increase in the pleasure derived from that outcome (and vice versa) [21]. BAS_drive_ has accordingly been used as a measure of trait reward-seeking in other studies of cognitive phenomena [28], [29], [30].

The distinction between BAS_drive_ and BAS_reward responsiveness_ has a parallel in the incentive salience hypothesis [1]. Berridge and Robinson describe an animal as ‘liking’ a reward when they have a strong hedonic experience, but ‘wanting’ a reward when they are driven to behave in a manner that will result in its consumption. Importantly, the incentive salience hypothesis proposes that the primary cognitive responsibility of dopamine is the creation of ‘wanting’, not ‘liking’. Given that BAS_drive_ appears to index much the same underlying construct as ‘wanting’, we approached the current experiment with particular interest in the BAS_drive_ subscale.

## Methods

### Participants

Thirty-seven neurologically typical students of the Vrije Universiteit Amsterdam gave informed consent before participation. Data from one participant was discarded due to high error rate (>2 standard deviations from the mean). Data from an additional participant was discarded due to incorrect completion of the BIS/BAS inventory. This participant made the same response to all but the first two of the inventory items and responded to many of the items with speeds inconsistent with the questions having been read or adequately considered. Two of the remaining 35 participants (6 men; age 20.4+/−2.3 years, mean +/− *SD*) were left-handed. All participants were paid for their participation.

All research was approved by the Vrije Universiteit Faculty of Psychology ethics board and conducted according to the principles of the Declaration of Helsinki.

### Experimental stimuli and procedure

The experiment took place in a sound-attenuated room and all stimuli were presented to participants via a CRT monitor located 60 cm from the eyes. The experiment began with completion of a computerized version of the Dutch translation of the BIS/BAS inventory [31]. This was followed by detailed instructions regarding the visual search task.

The search task was very similar to that employed in prior studies [13], [14], with the addition of reward feedback at the end of every trial (see Figure 1). Participants viewed stimulus arrays consisting of 10 shapes presented in a circle formation. Each shape was 9.1° of visual angle away from a central fixation point and 5.6° away from each of its two neighboring stimuli. The shapes were unfilled diamonds (4.2°×4.2°) and circles (1.7° radius) outlined thinly (0.3°) in red or green. A gray line (0.3°×1.5°) that could be randomly oriented either vertically or horizontally was presented in the center of each item.

The color and shape of the 10 stimuli were pseudo-randomly varied within the following confines. In each trial one of the objects was different in shape from the other nine. This could mean that a diamond was presented among circles or that a circle was presented among diamonds. In a quarter of trials this shape singleton was the only unique stimulus in the display, but in the remaining trials an additional singleton was defined by giving one of the identically shaped objects unique color (either red when everything else was green or vice versa). All stimuli were presented on a black background. Stimuli locations were randomized with the sole confine that the shape singleton could not also be of unique color.

Participants completed 30 blocks of 30 trials, which took approximately one hour. Each trial began with the presentation of a fixation point for a duration of 400 to 1400 ms followed by the presentation of a visual search array. Participant response was based on orientation of the line contained within the shape singleton; instructions were to press the ‘z’ key on a standard computer keyboard with their left index finger when the target line was vertical and the ‘m’ key with their right index finger when the target line was horizontal, and to do so as quickly as possible while maintaining an average accuracy of 90% or better. Feedback regarding accuracy and response latency was provided at the end of each experimental block. Participants were instructed to maintain eye fixation throughout the experiment and informed that eye movements were being periodically monitored via closed circuit camera. Correct responses to the search target were immediately followed by the replacement of the central fixation dot with an indication of reward feedback in blue text (65 point font; 5° height), either ‘+10’, denoting the receipt of 10 points, or ‘+1’, denoting the receipt of 1 point. The visual search display remained onscreen during the presentation of feedback and the search display and feedback were presented together for 1000 ms. Each point had a value of ∼0.2 euro cents and participants were paid based on the number of points they received. Because reward magnitude was random, the only performance factor that impacted earnings was accuracy. In the majority of participants accuracy was excellent, meaning that there was very little variability in pay: all subjects received 8 euro or more for participation.

## Results

### BIS/BAS scale

The BIS/BAS inventory results were consistent with those reported in other studies ([21], [29], [30]; mean BIS: 19.49+/−4.57 SD; BAS_total_: 13.84+/−1.624; BAS_drive_: 12.23+/−1.83; BAS_fun_: 11.40+/−2.14; BAS_reward_: 17.89+/−2.25). The BIS and BAS are theoretically orthogonal constructs [23] and prior results indicate that there is little in the way of a reliable relationship between measured BIS and measured BAS [21]. Our results were consistent with this; BIS correlated −0.157 with BAS_total_. BAS subscales showed a stronger relationship (BAS_drive_ to BAS_fun seeking_ 0.305; BAS_drive_ to BAS_reward responsiveness_ 0.546; BAS_fun seeking_ to BAS_reward responsiveness_ 0.241). Note that correlation values here and throughout this paper reflect Spearman's ρ, which is less sensitive to outlier values than other measures of correlation.

### Visual Search

Consistent with earlier research [13], participants were slower (840 ms vs. 787 ms; *t*(34) = 9.00, *p*<0.001) when the distractor singleton was present in the display (accuracy: 96.3% vs. 96.6%, *t*(34) = 1.08, n.s.).

As described in the Introduction, we were interested in comparing trials in which the target and distractor colors were either the same as they had been in the immediately preceding trial or had swapped. We limited analysis to trials in which the distractor was present in the display and response in the immediately preceding trial had been correct. Given that novel singletons [32] and new objects [33] will disrupt search to the greatest degree, we maximized our ability to detect the impact of the salient distractor by only analyzing trials in which the salient distractor was absent in the preceding trial. Furthermore, based on the observation of large costs related to response switch in this paradigm, we analyzed trials in which the response had not changed from the previous trial. A total of 9.5% of the selected trials were excluded from RT analysis: 5.1% because they were excessively slow (>1600 ms) and 4.6% because they resulted in incorrect response. This already-low error rate stemmed from a handful of participants; many participants made next-to-no errors in the experiment.

As illustrated in Figure 2, the receipt of high-magnitude reward caused strong priming: participants were fast to respond when the previous trial garnered high-magnitude reward and the color defining the current target was the same as that of the target in the previous trial. In contrast, participants were slow when the previous trial garnered high-magnitude reward and the target color in the previous trial now came to define the distractor. This pattern was statistically assessed in a repeated-measures analysis of variance (RANOVA) with factors for Color Swap (same vs. swap) and Reward Magnitude (low vs. high). This revealed a marginally significant main effect of Color Swap (*F*(1,34) = 3.158, *p* = 0.085, η^2^_p_ = 0.085), no effect of Reward Magnitude (*F*<1), and an interaction between the factors (*F*(1,34) = 4.944, *p* = 0.034, η^2^_p_ = 0.127). This replicates the central behavioral finding of our earlier work [11].

### Correlation of BIS/BAS to search behaviour

Our primary interest was in the relationship between trait reward-seeking - as measured by BAS_drive_ - and the priming of visual search by reward. Our expectation was that subjects with a high BAS_drive_ score would show a larger reward priming effect. In order to test this hypothesis, per-subject BIS, BAS_total_, BAS_drive_, BAS_fun seeking_, and BAS_reward_ scores were correlated with the priming effects observed in each of the low-magnitude and high-magnitude reward conditions. Priming effects were computed for each of the high-magnitude and low-magnitude reward conditions separately by subtracting the RTs observed in the no-swap condition from those in the swap condition. Results from this analysis, as illustrated in Table 1, revealed a strong and reliable correlation between BAS_drive_ and the magnitude of intertrial priming in the high-magnitude reward condition (ρ = 0.476, *p* = 0.004). No other correlations approached significance (all *p*s>0.3). Note that correlation statistics are based on comparison of the observed correlation to the distribution of correlation values garnered by relating all random permutations of the data.

**Table 1:** Correlations between BIS/BAS subscales and the impact of reward on intertrial priming in each of the low-magnitude and high-magnitude reward conditions.

	*BAS_total_*	*BAS_drive_*	*BAS_fun_*	*BAS_reward_*	*BIS*
*Low-magnitude reward*	0.119	0.116	0.065	0.175	−0.138
*High-magnitude reward*	0.244	0.476 *	0.114	0.168	0.056

*p<0.05.

**Figure 3:**
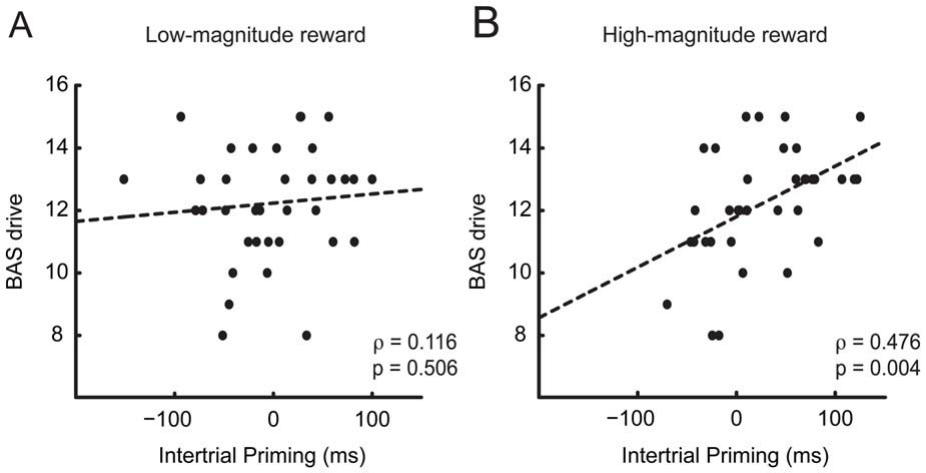
Per-subject scatter plots. a.) BAS_drive_ against intertrial priming observed in trials following low-magnitude reward, and b.) BAS_drive_ against intertrial priming observed in trials following high-magnitude reward. Linear fits of the data are denoted by broken lines.

Scatter plots of BAS_drive_ against intertrial priming for each of the low-magnitude and high-magnitude reward conditions are presented in Figure 3. Statistical analysis revealed that the difference between these correlations was marginally significant (*t*(34) = 1.616, *p* = 0.058 [34]). This is consistent with the idea that the relationship between BAS_drive_ and priming is exclusive to high-magnitude reward; participants who score high on BAS_drive_ do not generally show a larger intertrial priming effect, but a specific increase in reward priming.

## Discussion

This study was motivated by the idea that participants who are sensitive to the motivational valence of high-magnitude reward – who are reward-seeking – would be more likely to attend to stimuli characterized by reward-associated perceptual features and thus would show a larger reward priming effect. We measured trait reward sensitivity using the BIS/BAS inventory [21] and found that the BAS_drive_ subscale, a measure of the degree to which reward drives behavior, correlated strongly with the magnitude of reward priming observed in the visual search task.

These results add to a growing literature demonstrating the importance of reward to attentional control and attentional learning [7], [8], [11], [12], [34]. Prior work has shown that the sustained effect of attentional suppression – negative priming – is only present following high-magnitude reward, suggesting that the attentional mechanism responsible for creating this lingering inhibition is effective only following positive outcome [7]. A subsequent study has shown that participants are a.) able to efficiently select stimuli that have been consistently associated with high-magnitude reward, but have difficulty ignoring these objects, and b.) are able to ignore distractor stimuli when doing so in prior experience has resulted in a good outcome, but have trouble selecting these items when they are targets [8]. It seems likely that the pattern of results in this latter study reflects the operation of the same attentional mechanism responsible for the reward priming effect described here. Moreover, the perceptual and attentional benefits stemming from reward have been shown to have an impact on stimulus detectability, making reward-associated stimuli less sensitive to the attentional blink [35]. The current study is consistent with this developing literature, but also motivates a new perspective on these results: many of these effects may be subject to individual differences stemming from personality traits like reward-seeking.

This study also adds to another developing field investigating the influence of personality traits on perception and attention. To date, much of this work has focused on trait anxiety and the attentive response to threatening stimuli. There is substantial evidence that highly-anxious people attend to fear-related stimuli for longer than less anxious people and have difficulty disengaging attention from these stimuli [36]. Even more interesting are results suggesting that perceptual processing of fear-related stimuli is facilitated in high-anxiety individuals. For example, work with the visual search paradigm has demonstrated that participants who are afraid of spiders, but not snakes, will detect spiders more quickly than snakes, whereas participants who are afraid of snakes, but not spiders, will detect snakes more quickly than spiders [37]. In event-related potential work, facilitated processing of threatening stimuli in high-anxiety individuals has been demonstrated as early as the visual N1, which occurs 100 ms post-stimulus [38]. In conjunction with our earlier work [11 12], the present results suggest that trait reward-sensitivity can have a similar impact on early visual mechanisms, facilitating processing of reward-associated stimuli.
